# Neural network analysis of quasistationary magnetic fields in microcoils driven by short laser pulses

**DOI:** 10.1038/s41598-022-17202-2

**Published:** 2022-08-12

**Authors:** Iu. V. Kochetkov, N. D. Bukharskii, M. Ehret, Y. Abe, K. F. F. Law, V. Ospina-Bohorquez, J. J. Santos, S. Fujioka, G. Schaumann, B. Zielbauer, A. Kuznetsov, Ph. Korneev

**Affiliations:** 1 National Research Nuclear University MEPhI, Moscow, Russian Federation; 2 Centre Lasers Intenses et Applications (CELIA), UMR 5107, Université de Bordeaux - CNRS - CEA, Talence, France; 3 Institut für Kernphysik, Technische Universität Darmstadt, Darmstadt, Germany; 4 Institute of Laser Engineering, Osaka University, 2-6 Yamadaoka, Suita, Osaka 565-0871 Japan; 5 Graduate School of Engineering, Osaka University, 2-1 Yamadaoka, Suita, Osaka 565-0871 Japan; 6 Universidad de Salamanca, Salamanca, Spain; 7 PP/PHELIX, GSI, Darmstadt, Germany; 8 Lebedev Physical Institute, Moscow, Russian Federation

**Keywords:** Plasma physics, Applied physics

## Abstract

Optical generation of kilo-tesla scale magnetic fields enables prospective technologies and fundamental studies with unprecedentedly high magnetic field energy density. A question is the optimal configuration of proposed setups, where plenty of physical phenomena accompany the generation and complicate both theoretical studies and experimental realizations. Short laser drivers seem more suitable in many applications, though the process is tangled by an intrinsic transient nature. In this work, an artificial neural network is engaged for unravelling main features of the magnetic field excited with a picosecond laser pulse. The trained neural network acquires an ability to read the magnetic field values from experimental data, extremely facilitating interpretation of the experimental results. The conclusion is that the short sub-picosecond laser pulse may generate a quasi-stationary magnetic field structure living on a hundred picosecond time scale, when the induced current forms a closed circuit.

## Conversion of laser light to magnetic field in laser-driven coils

Strong magnetic fields affect properties of matter on different scales^1^. In the Universe, they may reach the Schwinger limit ruling extremely energetic astrophysical processes, while being very modest though necessary for life on Earth. Magnetic fields of a certain strength for high-end laser-plasma applications are routinely produced in laboratories, either with high-voltage discharge drivers, or in an optical way, meaning that the electric currents are induced by intense laser pulses. Indeed, invention of the Chirped Pulse Amplification technique^2^ potentiates laser radiation to possess an ultrahigh energy density and to become an excellent driver for strong electrical currents suitable for generation of extremely strong pulsed magnetic fields.

Several key approaches are extensively used for optical magnetic field generation (OMFG). The idea to use intense laser pulses for strong magnetic fields generation^3^ followed observations of intense currents and spontaneous magnetic fields in experiments of laser interaction with matter. Since the first experiments with specifically designed targets^4^ the optical approach is considered as a promising and convenient method. Generally, it is based on inducing electric currents in targets with certain loop-like geometries, when an intense laser pulse interacts with another part of the target. These so called capacitor-coil targets with a mm-scale size work in a quasi-stationary regime^5,6^, the strong electron current forms a closed circuit exciting and sustaining a strong magnetic field near the target loop. A wide range of possible applications is foreseen for this scheme, e.g. controlling high-energy charged particles transport^7,8^, enhancing fusion output in experiments on laser-driven implosion of magnetized inertial confinement fusion targets^9–11^ or producing magnetized plasma for laboratory studies of astrophysical processes^12–14^. Their compact size, no need of bulky and expensive capacitor banks and the ability to create magnetic fields one or two orders of magnitude higher than those reached with other methods make laser-driven generators preferable in many cases. Besides, various applications may require magnetic fields of different strength, geometry and temporal dependence, which may be effectively controlled by the parameters of laser pulses and the target.

Use of short laser pulses changes qualitatively the physics of magnetic field generation in capacitor-coil targets. The discharge may evolve rapidly and the setup needs optimization for efficient work. Intense short laser pulses are rather suitable for generation of energetic particles and secondary radiation, and they may also be more efficient for creating strong discharge electric currents, but just target size and setup down-scaling looks like an undesirable solution which limits many possible applications. Here, we show the way to reach a quasi-stationary operating regime in sub-millimeter targets with use of intense picosecond laser pulses.

When a short laser pulse interacts with an extended target, a short discharge pulse induced by the interaction propagates along the target^15^ almost with the speed of light. To reach a quasi-stationary regime with short laser pulses, reduction of the target perimeter down to the values less than the laser pulse length was proposed earlier^16–18^. A reduced size of the coil in OMFG makes the effective magnetized volume quite small, so the principal question addressed here is whether the target with the coil perimeter longer than the pulse length may produce a quasi-stationary magnetic field. In the presented experimental study, a coil-shaped target with the diameter $d\sim 100~ \upmu$m is irradiated on the free end. For this size the time needed for a discharge pulse to close the circuit is $\approx \pi d /c\sim 1$ ps, which appears to be longer than the 0.5 ps of the driver used. However, as shown below, if the circuit is closed before the discharge reaches the end of the coil, the generated magnetic field evolves towards stationary distribution. This allows to abstain from reducing the target size and shows the way of using powerful short laser pulses with practically interesting sub-millimeter targets.

**Figure 1:**
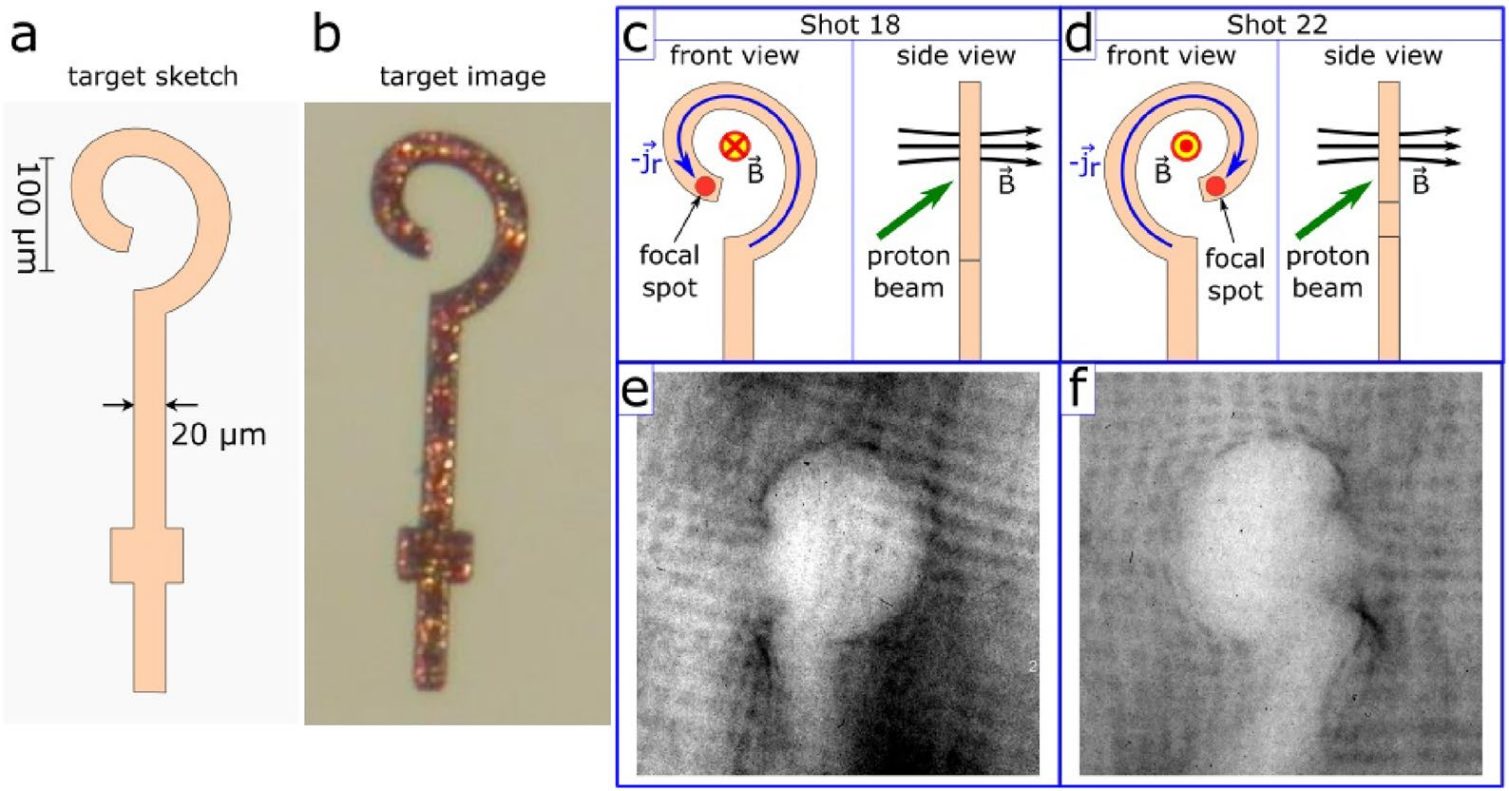
The target sketch (**a**) and the magnified photographic image (**b**). Scheme of the setup used in Shot #18 (**c**) and #22 (**d**). Proton radiography image obtained in Shot #18 in the second layer of RCF stack, corresponding to Bragg peak position for $\approx 3$ MeV protons, passing the studied region $\approx 25$ ps after the end of the laser pulse; darker colors correspond to higher proton concentrations (**e**). The same for Shot #22 (**f**).

**Figure 2:**
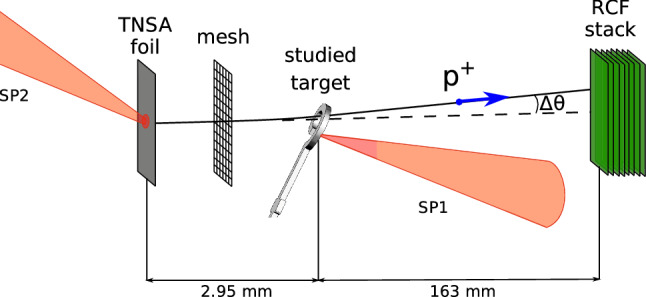
Sketch of the experimental setup for magnetic field generation and proton radiography measurements with two laser beams—SP1 and SP2.

The considered optically-driven magnetic field generator is a coil-shaped target, cut from $20 ~ \upmu$m copper foil, shown in Fig. 1a,b. The laser beam is focused on the free end of the coil, as shown in Figs.  1 and 2. Under the irradiation, the hot electrons escape the target^19,20^ inducing a strong positive potential, which drives a discharge current along the target. As the laser pulse length is shorter than the coil perimeter, the discharge forms a finite pulse, which would go to the ground for an open circuit. However, as shown below, for a reasonable thin slit, plasma from the irradiated coil end fills it before the front of the discharge pulse comes. This closes the circuit and allows the current to form a self-consistent quasi-stationary structure with the magnetic field. In the experiment presented here, the field was measured with proton radiography diagnostics^21–23^. In our study, the auxiliary protons generated with Target-Normal Sheath Acceleration (TNSA) mechanism^24^ are passing through the magnetized region and deflecting there according to the local fields, leaving afterwards an imprint on the radiochromic films, see Fig. 1 and Supplementary Material for further insight. To ensure the presence of the magnetic fields for a long time after the irradiation, the probing time reached a few tens of picoseconds.

## Experiment

Experimental study of optical magnetic field generation with coil-shaped targets was performed at PHELIX laser facility in Darmstadt, Germany. Laser-driven proton radiography was used as the main diagnostic for magnetic fields in the target region. The PHELIX laser pulse with wavelength of 1056 nm and duration of 0.5 ps was divided into two beams—SP1 and SP2, each containing an energy of $\approx 50$ J. The beams were tightly focused to a spot of $\approx 10 ~ \upmu$m FWHM (full width at half maximum) using two parabolic mirrors with focal lengths of 400 mm (SP1) and 1500 mm (SP2), yielding relativistic intensities $\approx 10^{19}$ W/cm$^2$ on the targets. SP1 was focused on the open end of the coil target to excite there strong discharge currents, while SP2 irradiated a thin gold foil, used as a source of diagnostic TNSA-accelerated protons^24^, which then passed through the induced electric and magnetic fields near the coil target, see Fig. 2. An imprint of the fields, deviating the protons, was collected by a stack of several HD-V2 radiochromic films (RCF). The active layer of each RCF colorizes under exposure to ionizing radiation, producing a proton image that contains information about the fields induced around the target. Multi-layer RCF setup enables to characterize their time evolution, since due to Bragg peak absorption of ions in matter each layer registers predominantly protons of one narrow energy range^25^, passing the studied region at a certain moment of time. The coil target was probed at ${27}^\circ$ to its axis, the distance between TNSA foil and the center of the coil target was 2.95 mm, the distance between the coil target center and the RCF plane was 163 mm, which corresponds to magnification factor of 56. Diagnostic protons had energies in the range of 1–6 MeV, yielding a time-of-flight difference of  130 ps for the specified distance between the target and proton source, and the time resolution of $<20$ ps, if the signal on the second and subsequent layers of the RCF stack, corresponding to $>3$ MeV protons, is considered. In order to distinguish diagnostic protons from protons emitted by the studied target, a metallic mesh with 1500 bars per inch was placed on the way of the probing proton beam.

Probing the generated fields in the experiment was performed for two opposing target orientations in shots #18 and #22, see Fig. 1c,d and the obtained radiography images in Fig. 1e,f. Their structure presents a void with distinct caustics on the boundary and a shadow of the target stalk. These images correspond to the latest time moment available, that is $\approx 25$ ps after the end of the laser pulse, which is much greater than its duration 0.5 ps. The considered layer possesses enough quality for the assessment of electromagnetic fields, while the signal on radiographs related to other time moments is not suitable for an accurate analysis: for earlier times the signal appeared to be rather dim, while for later times it was over-saturated. An interested reader can find these radiographs and additional explanations in the Supplementary Material.

Analysis of the obtained radiographs is a non-trivial inverse problem. A viable and commonly used approach is to perform the data assessment by comparison of experimental radiographs with those obtained in synthetic ballistic simulations, where probe protons pass through model magnetic and electric fields. Parameters of the model fields are adjusted to match the radiographs. With some assumptions, it is possible^5,6^ to estimate magnetic fields using only few geometric parameters of radiograph images, such as the width of the ’bulb’ region with the reduced proton signal. However, geometric fitting is not always possible, as the chosen parameters may depend similarly on both magnetic and electric fields, simultaneously present around the target. Therefore, analysis of the whole image is more robust as it may catch the entire structure formed by electromagnetic fields, rather than certain geometric features. In general, deformation of the imprint from a rectangular mesh that is placed in the way of probe protons before they pass through the fields may also be considered for analysis^5^, though, for short laser pulses, it may be blurred because of the transient processes of the field formation, as it is in Fig. 1e,f.

### Neural Network application to experimental data analysis

Nowadays, machine learning (ML) comprises of a wide range of computing algorithms, methods and approaches, that can successfully be employed for data-processing tasks in a broad range of research fields, including elementary particle physics and cosmology, quantum many-body physics, quantum computing, chemical and material physics^26^. One of the subsets of ML are artificial neural networks (ANN), which process data with an algorithm, ’trained’ in advance on completely characterized data sets. This approach can reduce computational costs and human workload in tasks involving large extensive data sets, e.g. the separation of pulsar signals from radio frequency interference^27^. Neural network-based methods can be used to make predictions without solving computationally costly equations for certain physical processes, e.g. dissolution kinetics of silicate glasses^28^ and turbulence in subsonic flows^29^. Image processing is one of the most known field of ANN applications. It is worth to note their use in image restoration in regular^30^ and fluorescent microscopy^31^, nonmodel-based bioluminescence tomography reconstruction^32^, rapid decoding of the sample image from its hologram over an extended depth of field range^33^, wavefront estimation^34^ or robust photomask synthesis in inverse lithography technology^35^. Recently it has been demonstrated using synthetic data, that ANN in principle can be used to analyse proton radiography images and deduce important magnetic field parameters^36^. Here we develop this idea to reconstruct electromagnetic fields in a real experimental setup using a Convolutional Neural Network (CNN), trained on numerically generated data. The chosen CNN architecture is known as one of the most widely used in various computer vision tasks^37^, and its modifications always win in annual ImageNet Large Scale Visual Recognition Challenge^38^. CNN possesses a significant advantage for our problem compared to other methods as it is highly tolerant to shifts of the region of interest inside the image. This benefit comes from the inclusion of one or several convolution layers that are able to extract some characteristic features of the image regardless of their position.

In order to produce radiographs in ballistic simulations and to create synthetic data for the ANN training, model electromagnetic field distributions are required. For that, 2D Particle-in-Cell (PIC) calculations were performed using codes Smilei^39^ and PICLS^40^. According to the results, detailed below and in the Supplementary Materials, the distribution of magnetic field in the target is mostly defined by surface electric currents. In the setup considered here, the electric currents are strongly confined by the target geometry and description of their evolution allows 2D consideration, while the laser-target interaction may need more sophisticated approaches. Due to the planar target geometry the currents are also assumed to have planar structure, with $J_x$ and $J_y$ being the main current components. Thus, in order to obtain magnetic field distributions is space, one can consider planar current loop consistent with 2D simulation results, orient it in accordance with the target orientation relative to the probe beam (see Fig. 2) and apply the Biot-Savart law to obtain the values of quasistationary magnetic field spatial distribution. Therefore, the first key parameter to model magnetic fields is the current value ${\mathcal {J}}$ along the target coil, closed through the expanded plasma near the irradiated coil end. Electric field distribution for ballistic simulations is defined by the target potential $\varPhi$, which is the second key parameter.

**Figure 3:**
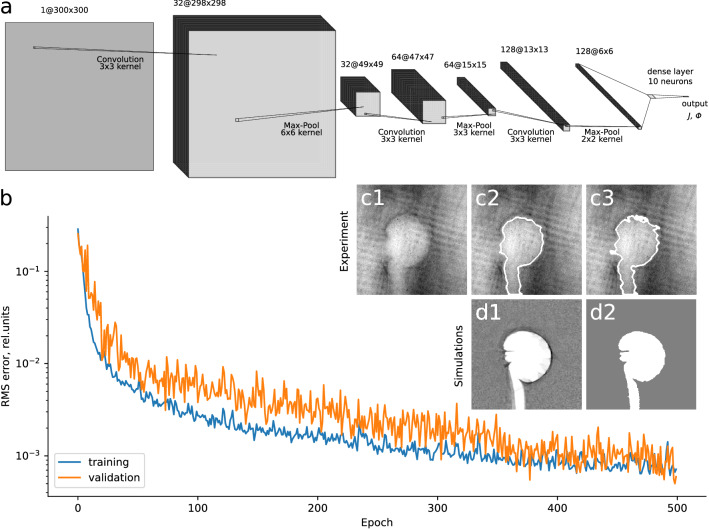
Architecture of the developed CNN (**a**) and learning curves for training and validation data sets, obtained in training run #1 and displaying the decrease of the mean squared error with the number of epoch (**b**). Panels (**c1**) and (**d1**) provide a comparison of proton patterns obtained in the experiment and in simulation for the field parameters extracted in training run #1. Corresponding ’void’ contours used for assessment of the fields are shown in panels (**c2**, **c3**, **d2**); for the experimental image two different possible contours are shown, (**c2**) corresponds to the parameters of the contour retrieval algorithm that intuitively provide a better fit of the ’void’ region boundary while (**c3**) corresponds to the parameters which match those that were used to retrieve the contours from all synthetic images. For easier comparison experimental contours are shown without inner fill and are imposed on the original experimental image.

For analysis, a CNN architecture was used, as described in the Methods section, see Fig. 3a. It allows detecting similar ’informative’ patterns in different parts of the data array^41^ and thus enables extracting desired parameters for images where the main void structure may be shifted or tilted. It was found, that the main information about electromagnetic fields near the target is encrypted in geometry of the ’void’ structure, which allowed to simplify the data and to reduce processing errors by using just the contour of the voids extracted from binarized images using OpenCV library tools (see Fig. 3, c2, c3, d2). This approach is not very sensitive to the quality of the proton beam, and the degree of colorization of a particular area of the RCF does not necessary have to be directly proportional to the number of proton that deposit their energy in this area. Such method is particularly relevant due to the fact that in the real experiment other sources of ionization were present—non-diagnostic protons from the studied target e.g. those with higher energies, fast electrons, X-ray and gamma radiation. Their interaction with the RCF stack could, for example, lead to some colorization of the inner ’void’ region, which is not reproduced in ballistic simulations. However, the shape of the ’void’ contours is defined mainly by the electromagnetic fields at the studied region and is not affected by the quality of the diagnostic proton beam and other factors. Thus, it is more appropriate to retrieve the field parameters from the images of the ’void’ contours.

In practice, there is an intermediate step needed to extract the contour from the whole image. Its shape depends on the parameters of the contour retrieval algorithm, which is detailed in the Supplementary Materials. In this work, the parameters are chosen so that on the one hand, no important information about the ’void’ shape is lost due to e.g. over-blurring and on the other hand, no artefacts of the proton beam or shadows of the metallic grid modify the main structure, defined by the examined fields, which act on diagnostic protons integrally along the whole trajectory causing therefore their regular deviations. The obtained parameter ranges appear to be wider for images with higher quality. For the experimental image, the exemplary contours extracted using parameters within a reasonable range are shown in Fig. 3, panels c2 and c3. For high quality synthetic data the contours are almost insensitive to the parameters in a reasonable range. So, the span of parameters, used in the intermediate step for the experimental data, defines an additional error of the fields definition. The parameters of the magnetic and electric fields presented below correspond to the contour shown in Fig. 3, c2, while other possible contours, shown in the Supplementary Material, are used to estimate the errors originated on this step.

‘Void’ contour images are the input to the CNN, while the two key field parameters ${\mathcal {J}}$ and $\varPhi$ are the outputs. The CNN is trained to recreate these continuous parameters, making it a regression problem in the established terminology. To create the pool from which the training and validation data can be drawn, 961 synthetic radiographic images with resolution of $300 \times 300$ were generated on a map $({\mathcal {J}}, \varPhi )$ with the total electric current ${\mathcal {J}} \in [2,50]$ kA range with a step of 1.6 kA (the magnetic field in the coil center ${\mathcal {B}} \in [25.3, 632.5]$ T range with a step of $\approx 19.6$ T) and the electric potential $\varPhi \in [0,150]$ kV range with a step of 5 kV. Before each image was passed into the ANN, slight random tilts and shifts were additionally introduced to it to make the ANN more robust and insensitive to the presence of these factors in the experimental data. A total of 10 training runs were performed. In order to split the data between the training and validation subsets, k-fold cross-validation^42^ with $k=10$ was employed as the resampling technique. For this purpose the whole data set was randomly divided into 10 subsets, one of which was selected for estimating the model performance while the rest were used explicitly to fit the model. Repeated training runs were performed with a different selection of the training and validation subsets, until all possible variants are exploited. The model performance was estimated as the average of the performance estimates on each of the training runs. The latter, in turn, was found by calculating root-mean-square deviations $\sigma _B^s$ and $\sigma _U^s$ of the predicted values for the synthetic data from their real numbers, available for the artificially created data. Hyperparameter values of the ANN such as the number of convolutional layers, number of filters, number of hidden nodes in the fully-connected layer and etc. were adjusted on the basis of grid search algorithm^43^, i.e. searching for the minimal value of the root-mean-square error for the validation set in the hyperparameter space of the learning algorithm. This method is one of the most simple and widely used ones along with the random search^44^. More sophisticated methods, see, e.g.,^45^, may be implemented in future researches to further boost the ANN performance. For example, typical learning curves corresponding to training run #1 and displaying the decrease of the mean squared error with the number of epoch are shown in Fig. 3b. The training is halted after 500 epochs, although the validation curve still continues to decrease, because prediction errors for the synthetic data at this point become sufficiently low and are not expected to change dramatically with additional training. The trained models were then applied to the real experimental data and a total of $k=10$ predictions for the magnetic field at the coil centre $B_{pred.}$ and electric potential of the target $U_{pred.}$ were obtained. The results of 10 training runs including the root-mean-square spread $\sigma _B^s$ and $\sigma _U^s$ obtained with the synthetic data, predictions $B_{pred.}$ and $U_{pred.}$ made on the real experimental data, as well as their average values and root-mean-square deviations $\Delta _{RMS}$ of $B_{pred.}$ and $U_{pred.}$ from their mean are summarized in Table 1.

**Table 1:** Summary of the results obtained by the ANN in 10 training runs: root-mean-square errors $\sigma _B^s$ and $\sigma _U^s$ obtained using the synthetic data, and predicted values for the magnetic field at the coil center $B_{pred.}$ and the electric potential of the target $U_{pred.}$, obtained by passing the real experimental image through the trained CNN.

Run #	$\sigma _B^s$, T	$\sigma _U^s$, kV	$B_{pred.}$, T	$U_{pred.}$, kV
1	7.8	1.7	235.9	30.2
2	12.4	2.0	209.4	35.8
3	10.4	2.3	205.5	30.3
4	9.1	1.7	215.3	28.9
5	12.2	2.4	187.2	36.5
6	9.0	2.0	198.0	35.0
7	8.2	2.1	229.6	33.8
8	8.3	1.6	234.0	24.4
9	9.6	2.4	223.4	30.3
10	10.9	1.7	183.8	38.7
Average	9.8	2.0	212.2	32.4
$\Delta _{RMS}$	–	–	17.8	4.1

As can be seen from Table 1, root-mean-square errors $\sigma _B^s$ and $\sigma _U^s$ are rather small, meaning the model performs quite well on the synthetic data. Namely, it can retrieve magnetic fields in range [25.3, 632.5] T with a root-mean-square error of about 10 T, which is less than 2% of the range size; and for the electric potential in range [0, 150] kV the root-mean-square error is just 2 kV, which is close to 1% of the range size. However, the actual error for the real experimental data is different. Each fitted model predicts slightly different values of the magnetic field and electric potential, resulting in some variability of the retrieved parameters. Thus, their average values are considered as final estimates obtained by the ANN-based approach, while root-mean-square deviations from these average values $\Delta _{RMS}$ characterise dispersion, or degree of variability of the ANN-based estimates when the ANN is applied to the real data. According to Table 1, the corresponding root-mean-square errors resulting from this variability amount to $\sigma ^B=8$% in case of the magnetic field at the coil centre and $\sigma ^U=13$% in case of the electric potential of the target. The resulting error can be estimated by multiplying the obtained standard deviations by the Student’s t-coefficients for a given confidence interval. For the confidence level of 95% we obtain relative errors $\delta _{tr.}^B \approx 18$% for the magnetic field and $\delta _{tr.}^U \approx 29$%. An additional source of error is related to the contour retrieval uncertainty. Complementary analysis performed with different possible experimental contour shapes provided in the Supplementary Material shows that the resulting overall spread of the values in root-mean-square error sense is about $\sigma _{cont.}^B = 6$% for $B_{pred.}$ and $\sigma _{cont.}^U = 16$% for $U_{pred.}$. The resulting errors for the same 95% confidence level are $\delta _{cont.}^B \approx 14$% and $\delta _{cont.}^U \approx 36$%. These errors are of the same order as the training uncertainty calculated from the results of multiple training runs, see Table 1, and thus both errors should be taken into account to assess the overall accuracy of the method. Estimating the total error as $\delta _{tot.}=\sqrt{\delta _{tr.}^2 + \delta _{cont.}^2}$, where $\delta _{tr.}$ denotes the error related to the ANN training and $\delta _{cont.}$ denotes the error resulting from the contour selection uncertainty, gives $\delta _{tot.}^B \approx 23$% and $\delta _{tot.}^U \approx 46$% for the relative errors of the magnetic field and the electric potential, respectively. Note that the relatively large values are the consequence of the high confidence level.

Two additional tests were conducted to justify the choice of the CNN architecture for this problem. In both cases all convolutional layers and the second and third pooling layers were removed. Thus, only the first pooling layer was kept in order to reduce the size of the data and the number of parameters for optimization. The rest of the hyperparameters were left untouched. In the first of the two tests synthetic data with no additional modifications, i.e. shifts and tilts, was used. In contrast, in the second test random shifts in range $\pm \, 20$% of the image size and random tilts in range $\pm \, 10^{\circ }$ were introduced to the images from both synthetic and validation data sets. It was found than in the first case the root-mean-square errors for the magnetic field and the electric potential are $\approx 15$ T and $\approx 3$ kV, respectively, which complies with the values obtained by using the CNN architecture (see Table 1). However, in the second case the errors increased by more than a factor of 3, up to 46 T for the magnetic field and 11 kV for the electric potential. Thus, we can conclude that convolutional layers do indeed play a significant role in the analysis of imperfectly positioned patterns, which is the case if real experimental data is considered. Therefore, it is appropriate to use the CNN for this problem.

**Figure 4:**
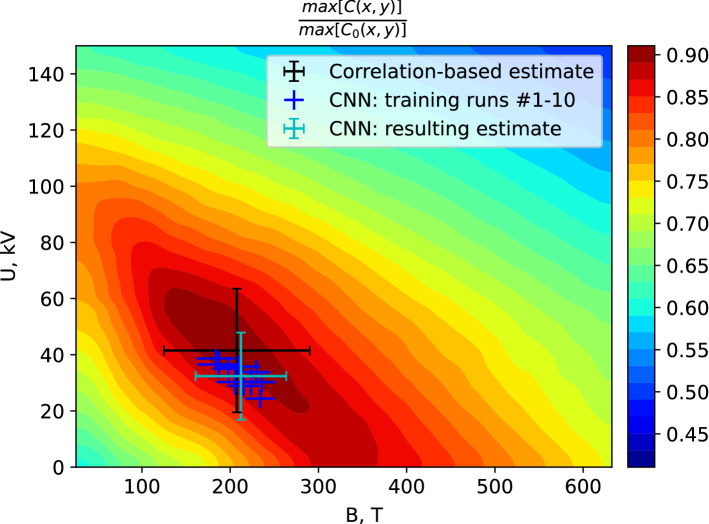
The peak value of cross-correlation between the normalised experimental and synthetic images as a function of the magnetic field in the target center and the electric potential of the target. The correlation peak values are normalized per maximum of the autocorrelation function $C_0(x,y)=\sum _{u,v}f(x,y)f(x+u,y+v)$. Black cross with error bars shows the best-fit for the region of the maximum cross-correlation peak value. Positions of the blue crosses show the ANN-retrieved results obtained by each of the 10 models that were fit and validated on different subsets of the data, while their size indicates retrieval errors estimated by testing the model on the validation subsets created from the artificially created data. Cyan cross shows the resulting CNN-based estimate obtained by taking the mean of 10 predictions made by the models trained on different training runs, see Table 1; its error bars correspond to the uncertainty which results from the spread of the predicted values and the contour retrieval error, with the size of the interval corresponding to the 95% confidence level.

The validity of the ANN-produced results was additionally studied with a correlation analysis. Two-dimensional cross-correlation functions were calculated for the pairs of the experimental image and the synthetic images on the map $({\mathcal {J}}, \varPhi )$ as $C(x,y)=\sum _{u,v}f(x,y)g(x+u,y+v)$, where *f* and *g* are the normalized pixel values of the experimental and synthetic images. The peak of the cross-correlation function was attributed to the image similarities. In Fig. 4 the distribution of the cross-correlation peak values are shown. The region of high correlation has the black cross in the center with error bars indicating deviation of the cross-correlation peak from its maximum for less than 0.02. As can be seen, there seems to be a sufficiently strong anti-correlation between the magnetic and the electric field. Increasing the one while the other remains the same may lead to similar results, as in the case when they are swapped. However, there are certain features that enable distinguishing between the two. One of them is the shadow of the stalk, the shape of which is mostly defined by the electric potential. It enables to constrain the value of the latter and determine the magnetic field using the rest of the structure. From this consideration, the values of $208 \pm 83$ T are obtained for the magnetic field and $\varPhi =42 \pm 22$ kV for the electric potential. The CNN-based results are shown in the same plot by blue crosses. They are scattered in the area shifted from the best-fit region in the correlation-based estimate. Although the stalk shadow is accounted for in the training data, the ANN-results also seem to exhibit some anti-correlation between the magnetic field and the electric field, implying that the constrained range for the latter still leaves some adjustment freedom, leading to the observed retrieval errors.

The results obtained with the two different methods closely coincide with one another, although some systematic difference is observed. It is related mostly to the potential $\varPhi$. So, it could be explained by the different treatment of a certain image feature related to the electric field. One such feature is the shadow of the target stalk, since the magnetic field in our consideration is mostly confined inside the target cavity. The size of the stalk shadow varies along the perimeter and it has no distinct borderline, see panels c1–c3 on Fig. 3. These irregularities may lead to a bias in the value of the extracted electric potential of the target observed.

A major advantage of the neural network-based method is its computational effectiveness in case of greater amount of experimental data. Initially, both methods used require a synthetic data set. With it, the neural network-based method requires a one-time computation of training the artificial neural network, while for the two-dimensional cross-correlation function calculation is necessary for each analysed image. The trained ANN without much additional efforts allows to obtain the result for the second experimental image in Fig. 1, (f), proposing the magnetic field value of $250 \pm 130$ T. Obviously, neural network-based method is definitely preferable in case of an extensive parametric scan for a certain OMFG scheme.

## Numerical particle-in-cell simulations

**Figure 5:**
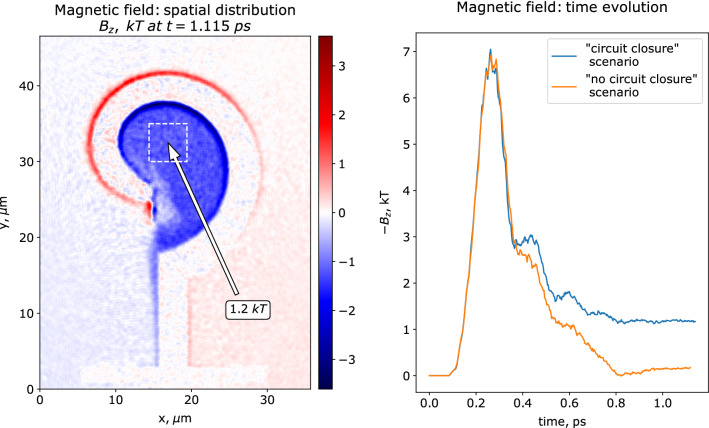
Results of 2D PIC simulations. Left panel: spatial distribution of magnetic field $\approx 0.75$ ps after the end of the laser pulse, averaged spatially with a Gaussian kernel of $\approx 0.5~\upmu$m to reduce visual noise. Right panel: time evolution of the magnetic field inside the ’snail’ cavity for two different scenarios of magnetic field generation, showing that the generated magnetic field evolves towards stationary distribution if the circuit is closed before the discharge reaches the end of the coil, which proposes a way of using short laser drivers with practically interesting large coils. Magnetic field is averaged over a $5 \times 5~\upmu$m$^2$ square, marked with the white dashed line on the spatial distribution plot.

Phenomenon of the magnetic field generation in the considered setup may be explained with a simple and transparent physical model. According to the numerical modelling, a quasi-stationary magnetic field is formed only if the current circuit closes through the laser-generated plasma. An insight comes from the parametric scan, performed with the reduced simulation setup. Targets in this set of simulations consist of electrons ($\approx 90$ times the critical density) and ions with charge $Z=10e$ and mass $A=544$ corresponding to $\times 8.5$ the atomic mass of copper, to equate mass densities to the real values. The simulation box is $35.7~\upmu$m$\times 46.5~\upmu$m or $5376 \times 7008$ cells with 10 particles of each kind per cell, the time step is $\approx 7.5 \cdot 10^{-3}$ fs. The laser intensity is $5.55\times 10^{19}$ W/cm$^2$. Magnetic field distribution at $\approx 1.1$ ps is shown in Fig. 5 (left panel), it is almost uniform with an average value of $\sim 1$ kT. The temporal behavior of the magnetic field in the coil in this simulation is shown with a blue curve in Fig. 5 (right panel). Following the fast growth up to $\sim 7$ kT at $\approx 0.25$ ps, the magnetic field gradually decreases after the laser pulse ends. However, at $\sim 0.8$ ps the decrease stops at $\sim 1$ kT level. A quasi-stationary field distribution is formed and stays then for a time, much exceeding the laser pulse duration. This happens if the circuit closes through the gap between the end of the coil and its opposite side due to expansion of the laser-heated plasma with density high enough to sustain the current in the coil. For magnetic fields of $\approx 1$ kT the surface current density is about 1 kA/$\upmu$m. A simple estimate where the required electron density $n_e$ in the gap is related to the surface current density *j* as $n_e={j}/{e w c}$, where $w \approx 2$ $\upmu$m is the width of the conducting layer in the gap, and *c* is the light velocity, gives $n_e\sim 10^{19}$ cm$^{-3}$. In simulations, the electron density in the gap reaches $10^{18}$–$10^{19}$ cm$^{-3}$, which is in a qualitative agreement with the obtained estimate. In contrast, if the circuit does not close before the laser-induced current pulse passes the coil, the magnetic field further decays to zero, as shown in Fig. 5 (right panel), with an orange curve. This situation was modeled by increasing artificially the mass of ions by 36 times, which sufficiently decreases plasma expansion, so that the electron density in the gap does not not exceed $10^{16}$ cm$^{-3}$ when the discharge passes the coil length. In this case the discharge then propagates further to the stalk. The numerical analysis is presented in more details in the Supplementary Materials.

In the experiment, the magnetic field deduced from proton measurements corresponds to the total electric current of $\approx 20$ kA. In order to evaluate electron density in the gap, estimate the number of electrons with the energy that obeys ponderomotive scaling $N_e=\chi E_{las} / (m_e c^2 \sqrt{(1 + \chi a_0^2 / 2)} - m_e c^2)$, where $\chi \sim 0.1..0.2$ is the laser absorption coefficient, $E_{las}=50$ J is the total energy in the laser beam, $m_e c^2 \approx 0.5$ MeV is the electron rest energy and $a_0 \approx 3.8$ is the normalized vector potential that corresponds to the laser intensity of $2 \cdot 10^{19}$ W/cm$^2$. The result $N_e \approx 10^{15}$ is distributed in the volume of $\approx 50^3$ $\upmu$m$^3$, sufficient to close the internal tip of the spiral and its opposite end, the value of $\sim 10^{20}$ cm$^{-3}$ is obtained for the average electron density in the gap. Multiplying it by a factor *eSv*, where $S=10 \ \upmu$m$^2$ is the area of a conducting layer and $v\approx c$ corresponds to the light velocity for relativistic electrons, we deduce the electric current $\approx 100$ kA, which is enough to explain the stationary behavior of magnetic field observed in the experiment.

## Outlook

Many foreseen optical-based applications, like particle acceleration, involve intense short laser pulses. For accelerated particle control, same laser pulses may create quasi-stationary electromagnetic structures, with a well controlled magnetic component, created by a closed current circuit in a coil. For potentially interesting big targets, the circuit closure may be controlled by the same laser driver, which is responsible for the excitation of the discharge current, even for very short laser pulses in femtosecond domain^46^. Important conclusion of the presented analysis is that *in the short laser pulse regime, the stationary structure formed by electric current with its magnetic field needs an electric connection between the two loop ends*. This relates to the coil targets considered here as well as to the capacitor-coil targets and their variations studied elsewhere. The connection may be formed by plasma expanded around the irradiated spot, and with it, the quasi-stationary models describing the current and magnetic field evolution may become valid. Otherwise, the relevant physical model is a non-stationary discharge pulse propagation along a curved wire, resulting in rapidly decreasing transient magnetic fields.

With use of the Neural Network, we deduce from the experimental data, obtained for a coil irradiated with a picosecond driver delivering several tens of Joules, that the magnetic field is of the order of hundreds of Tesla long after interaction. Namely, the magnetic field was estimated as $\approx 210 \pm 50$ T and $\approx 250 \pm 130$ T for the two opposite target orientations, about 25 ps after the end of the laser pulse. The field structure remains stationary for tens of picoseconds due to the discharge circuit closure, which is evidenced by theoretical modelling. The presented neural network-based method of retrieving the field parameters shows a good accuracy, robustness and higher computational effectiveness than an alternative correlation-based technique. Unlike the correlation analysis, it does not require additional interpolation between data points and in principle can be used to obtain electromagnetic field values outside of the ranges used to construct synthetic data sets, though presence of a systematic error may depend on the validity of the assumed electromagnetic field structure as well as the data quality.

## Methods

### Synthetic data generation with ballistic proton simulations

The training data was created in a set of ballistic simulations where Newton’s equations of motion for individual protons under the action of the Lorentz force were solved for different model magnetic and electric field distributions $d{\vec {p_i}}/dt=q_p {\vec {E}}({\vec {r_i}}, \varPhi ) + q_p [{\vec {V_i}} \times {\vec {B}}({\vec {r_i}}, {\mathcal {J}}) ]$, where ${\vec {r_i}}$, ${\vec {V_i}}$ and ${\vec {p_i}}$ describe the coordinates, velocity and momentum of a test proton with electric charge $q_p$ at a time moment *t*, while ${\vec {B}}({\vec {r_i}}, {\mathcal {J}})$ and ${\vec {E}}({\vec {r_i}}, \varPhi )$ are the quasi-stationary magnetic and electric fields at query points ${\vec{r_i}}$, proportional to the electric current in the loop ${\mathcal {J}}$ and the electric potential of the target $\varPhi$, respectively. The magnetic field was calculated with $10 ~\upmu$m resolution using the Bio-Savart law under the assumption that it is formed by discharge currents flowing along the coil inner and outer surface. Electric field was calculated on the same $10 ~\upmu$m grid under the assumption that the target is charged to a certain potential. Based on PIC simulations, it was concluded, that the change rate for the magnetic field during its measurement 25 ps after the end of the laser pulse was low in comparison to the time necessary for the particle to pass the studied region, i.e. magnetostatic approximation was made. And the electric field at the same time moment was mainly expected to arise from the positive charge formed on the target as a result the outer electron ionization. As this charge had enough time to redistribute with approximately the velocity of light across the whole target, the latter was assumed to be uniformly charged to a fixed potential and electrostatic approximation was used. Protons in ballistic simulations were considered to originate from a point source. Charge-separation effects in the proton beam were neglected, as they are typically relatively low in TNSA-generated particle beams^47^. Thus, proton deflection was assumed to be caused purely by electromagnetic fields induced around the target. The force acting on a proton was linearly interpolated from the values at the grid points. Time resolution was chosen in accordance with the grid step $\Delta$ and initial proton velocity $V_0$: $dt = \frac{\Delta}{2V_{0}}$. To account for a shadow produced in the beam by protons that hit the target material all particles with trajectories intersecting the target body were terminated from the simulation. Using such test particle approach, we created synthetic data sets for training and validating the neural network for magnetic field values at the target centre in the range from 25.3 T to 632.5 T and electric potential values in the range from 0 to 150 kV.

### Neural network architecture

For analysis of the obtained data, a CNN architecture was used, as it allows detecting similar ’informative’ patterns in different parts of the data array^41^ and thus enables to extract desired parameters for images where the main proton void structure may be shifted. It is especially important when working with both synthetic and experimental data jointly, since it allows to skip an alignment of simulated to real image data. The first stage of our network consists of three convolutional layers, each followed by a pooling layer. The former are used to produce a set of feature maps by convolving the input image with different optimizable kernels. Each feature map, obtained with one specific kernel, is then transmitted through a nonlinear activation function, for which we used a common Rectified Linear Unit (ReLU): $f(x)=max(0,x)$. The obtained data arrays undergo down-sampling in a pooling layer. This is achieved by dividing feature maps into small patches and taking local maxima as the new feature map ’pixel’ values. Pooling effectively reduces the size of the data and the number of parameters for optimization, while substituting several neighboring ’pixels’ with a single value makes ANN tolerant to small shifts and distortions in the analysed image. Output of the third pooling layer is flattened and sent to a fully-connected (dense) layer, connecting each node of the previous layer to every node of the subsequent final layer. It is composed of 10 neurons which take an input vector, apply to it optimizable weights and biases and pass it through an activation function. For this layer another common nonlinear function was used: $f(x)=\sigma (x)=(1+e^{-x})^{-1}$. The described architecture is illustrated in Fig. 3a. The output layer has two nodes, corresponding to the two aforementioned key variables the ANN is trained to extract. The process of training implies iterative optimization of feature extracting kernels along with weights and biases of each neuron. On each iteration (epoch) loss function is calculated, which in our case presents mean squared error of extracting the two key parameters. Then the kernels and weights in every layer are updated to minimize the loss function. It is performed on the basis of an optimization algorithm, in this particular instance first-order gradient based optimization employing Adam algorithm^48^ was used.

### Cross-correlation analysis

Correlation analysis was employed as an additional method of retrieving electromagnetic field parameters. In order to implement it, we took the same data set that was used for the training of our neural network. For each image from this data set a two-dimensional cross-correlation function was determined by computing the sliding dot product of the experimental image with the given synthetic one. This enables finding similar features in both images regardless of their position in the image. Thus, when the main patterns corresponding to the ’void’ structure overlap, the value of the cross-correlation function increases. The better the images overlap, the higher is their cross-correlation, making it a useful metric for determining the degree of similarity between two images. In our case, the maximum value of two dimensional cross-correlation was used. All the compared images were preprocessed—first, a mean pixel value was subtracted from each image and afterwards the image was divided by its standard deviation. This procedure enables to ensure that the increase of the cross-correlation peak is caused solely by the close overlap of the informative ’void’ structures with each other and is unaffected by high background noise or different brightness of one of the two images. The resultant correlation peak values were divided by the value of the autocorrelation peak, which implies comparison of the experimental image with itself, thus making 1.0 the highest degree of similarity in such consideration, and interpolated on a grid with resolution $\approx 2$ T for the magnetic field at the target centre and $\approx 0.5$ kV for the electric potential of the target. As a result, two-dimensional correlation maps with the distinct ’best-fit’ region with high correlation in a certain range of parameters were obtained, enabling a simple estimation of the parameters of the fields and their comparison to the results, obtained with the artificial neural network.

A two-dimensional plot in Fig. 4 shows the dependence of the value of cross-correlation peak on the magnetic field in the target center and the electric potential of the target. The correlation peak values are normalized per maximum of the autocorrelation function for which both *f* and *g=f* are pixel values of the same experimental image. The region of high correlation and, hence, the region in which the field parameters provide the best fit to experimental data, has a prolonged oval shape, with the black cross in the center.

In the case of complex field distributions the described ANN approach would be much more computationally effective in a long run. After creating the synthetic data, the neural network-based method would require a one-time computational cost of training the artificial neural network, while for the other method computation of two-dimensional cross-correlation function would be necessary for each new analysed image. Thus, neural network-based method is more preferable when there are multiple images for the same target which need to be analysed. If the distribution of electromagnetic fields is the same for these images, and only its parameters are different due to, for example, different laser intensity or target material, it would only take a few seconds to preprocess the images and pass them through the trained network to obtain the values of these parameters. In order to illustrate this idea, we have estimated the total computational time which would be necessary with our computational resources to process 6 experimental images, corresponding to the same target, but, for instance, 3 different laser intensity levels and 2 different target materials, for the case when the electromagnetic field distribution is parametrized by 3 values instead of 2, as it was in this paper. The resultant total time is estimated to be about 28 h with the neural network-based method and about 70 h with the cross-correlation analysis. Although, despite obvious computational advantage of the former, it should be noted that the correlation analysis allows to obtain a multi-dimensional map and estimate the size of the region where the parameters closely reproduce experimental ones, and thus properly estimate evaluation errors for these parameters. However, with a more complicated electromagnetic field structures, ANN-based approach would allow to explain the data in a multi-dimensional parameter space, where other methods, like a cross-correlation analysis, would take too much time.

## Supplementary Information

Supplementary Information.
