# Supplementary material for: Neural network analysis of quasistationary magnetic fields in microcoils driven by short laser pulses
Source: Sci Rep. 2022 Aug 12;12:13734. doi: 10.1038/s41598-022-17202-2 (PMC9374746; doi:10.1038/s41598-022-17202-2)
Supplement: Supplementary file 1 — Supplementary Information. [file 41598_2022_17202_MOESM1_ESM.pdf]

Supplementary Material
for
Neural network analysis of quasistationary magnetic fields in microcoils driven by short laser pulses

I. V. Kochetkov[1], N. D. Bukharskii[1], M. Ehret[2,3], Y. Abe[4,5], K. F. F. Law[4], V. Ospina-Bohorquez[6], J. J. Santos[2], S. Fujioka[4], G. Schaumann[3], B. Zielbauer[7], A. Kuznetsov[1], and Ph. Korneev[1,8,*]

[1]National Research Nuclear University MEPhI, Moscow, Russian Federation
[2]Université de Bordeaux - CNRS - CEA, Centre Lasers Intenses et Applications (CELIA) UMR 5107, Talence, France
[3]Institut für Kernphysik, Technische Universität Darmstadt, Germany
[4]Institute of Laser Engineering, Osaka University, 2-6 Yamadaoka, Suita, Osaka, 565-0871, Japan
[5]Graduate School of Engineering, Osaka University, 2-1 Yamadaoka, Suita, Osaka, 565-0871, Japan
[6]Universidad de Salamanca, Salamanca, Spain
[7]PP/PHELIX, GSI, Darmstadt, Germany
[8]Lebedev Physical Institute, Moscow, Russian Federation
[*]korneev@theor.mephi.ru

June 1, 2022

1 Particle-In-Cell simulations

Interaction of the PHELIX laser beam with the spiral target was extensively numerically modelled with the Particle-In-Cell codes SMILEI [1] and PICLS [2] in 2D geometry. Two sets of simulations were performed: (i) simulations with the target size and geometry, close to the real one in the experiment, (ii) simulations with the target geometry, close to the real one, and the target size reduced by a factor of ≈ 6. Simulations with the real target size and geometry, see Fig. S1 were performed using PICLS. The goal of these simulations was to prove that the main features of the magnetic field generation process do not depend on the actual way of heating the target, since in 2D the interaction geometry was different from the real experimental geometry. In these simulations, the target was made from singly charged electron-ion plasma, with heavy ions $m_i = 11750 m_e$, 50 particle per cell for both electrons and ions, electron density 10 times the critical density. Laser pulse was linearly polarized with intensity of 2×10^{19} W/cm^2 and duration of 0.5 ps. Laser pulse was modelled in two different ways in the two simulations. The first way corresponds to the laser pulse, propagating in the simulation plane, entering the simulation box from the left boundary, and heating the coil end, where a small tip was introduced to catch the pulse energy, see Fig. S1 a & b. The second way is imitating the laser pulse as coming into the simulation plane, the technique, described in details in [3]. In this case, the laser is focused on the coil end, so that the energy is effectively injected inside the target material, see Fig. S1 c & d.

The simulation results are presented in Fig. S1. For the laser pulse, injected from the left boundary, the two left columns shows for different times 0.49, 0.7, 1.1, 1.4, 1.8, 5.8 ps the magnetic field component B_z in panels a1-a6 and electron density in panels b1-b6. Just after the interaction, a discharge current pulse is excited and propagates along the coil from the irradiated end. In ≈ 1 ps, it reaches the opposite part of the coil and is divided in two parts, one coming to the stalk, and another passing through the expanded plasma passes the

gap and continues to go along the coil, see Fig. S1 a4 & b4. Due to a circuit closure at a time, when the discharge pulse reaches the end of the coil part of the target, it forms a continuous current in the coil, with a homogeneous magnetic field inside, see Fig. S1 a6. The magnetic field value of ≈ 500 T is qualitatively consistent with the experimental measurements. In case of the laser pulse, coming normally to the simulation plane, the two right columns in Fig. S1 shows for different times 1.11, 1.32, 1.67, 2.02, 2.38, 5.8 ps the magnetic field component B_z in panels c1-c6 and electron density in panels d1-d6. The scenario of the magnetic field formation is exactly the same as for the side irradiation geometry. The most important difference is a higher value of the discharge current and the magnetic field. This is explained by some overestimating of heating the plasma in the used model, which occurs in this case inside the target bulk, and increases the absorption efficiency. These two simulations show, that the exact absorption mechanism is not critically important for formation of the discharge current and the final magnetic field, supporting the use of 2D simulation geometry in the considered essentially 3D experimental setup. The most important is that the quasi-stationary formation of the magnetic field needs the circuit closure.

Simulations with the reduced target size and real geometry, see Fig. S2, were performed using the code Smilei. The purpose of this set of simulations was to study the effect of circuit closure and confirm its importance for the quasi-stationarity of the magnetic field generated in picosecond laser pulse regime. In the first simulation of this set the target was composed of electrons and ions with electric charge $Z = 10$ and atomic mass $A = 544$, corresponding to the atomic mass of copper, increased by a factor of 8.5 to compensate the decreased values of electron and ion densities in comparison to the solid-state values. For the second simulation the ion mass was additionally increased by a factor of 36. Each cell contained 10 macro-particles of each kind. The electron density was 90 times the critical plasma density. The linearly polarized laser pulse with the wavelength 1 μm, intensity of 5.55×10^{19} W/cm^2 and duration of 0.125 ps (FWHM) enters the simulation box from the left side and focuses to a spot with $1/e^2$ diameter of 3.2 μm on the tip of the coil, which was prolonged downwards to capture a reasonable part of the laser energy.

The results, obtained in this set of simulations, are presented in two columns in Fig. S2. The first column corresponds to the simulation with 'light' ions ($A = 544$), while the second one to the simulation with 'heavy' ions ($A = 19584$). In each column spatial distributions of magnetic field and electron density are shown for consecutive time moments covering different stages of the interaction. Initially, at the stage where the laser pulse induces a strong discharge current wave that starts propagating along the coil perimeter, both cases exhibit similar behaviour (see plots for magnetic field at times $0.121 - 0.362$ ps). At $t = 0.482$ ps the discharge wave for the most part has passed the coil perimeter and most of it goes down along the stalk. This is the moment when a distinction can be drawn between the results of the two simulations. As can be seen on the plots for the subsequent time moments (0.603 ps and 1.115 ps), the magnetic field in the left column, corresponding to 'circuit closure' scenario, decays to an average value of ≈ 1 kT inside the cavity, when the decay stops, while in the second column it reaches almost zero level, decaying into noisy background with only some traces of the structure that was present before the discharge current wave passed along the target stalk. Examining electron density plots for the two scenarios explains such behaviour. In the case of 'light' ions the plasma, formed near the target tip, expands and the electron density in the gap between the tip and the opposite end of the coil connected to the stalk rises to the level $10^{18} - 10^{19}$ cm^{-3}, which is sufficient to support the surface current density of 1 kA/μm yielding the magnetic field of 1 kT at the centre of the cavity. The mentioned values of electron density in the gap can be observed on the plot in the left panel of Fig. S3, which also corresponds to the spatial distribution of electron density at $t = 1.115$ ps, but with no spatial filtering applied. The case of 'heavy' ions corresponds to the opposite scenario, when the electron density in the gap is not sufficient to support high electric current and the inner magnetic field structure. As can be seen on the plots for electron density in the right column, initially, during the early stages of the interaction, some electrons leave the area of the target tip, but afterwards, when the current wave has passed the coil perimeter, most of them are confined with ions near the target tip. This leads to insufficient electron density in the gap and thus, to a fast decay of the magnetic field to an almost zero level. This set of simulations confirm the importance of the circuit closure for the generation of the long-living magnetic field structure with picosecond or subpicosecond laser pulses.

2 Overview of the experiment and the observed data

The experiment was conducted at the PetaWatt laser facility PHELIX, GSI, Darmstadt, Germany. Laser pulse at wavelength 1056 nm with a duration 0.5 ps was divided into two beams, SP1 and SP2, each containing ≈ 50 J of energy. These beams were directed into the vacuum chamber and focused at the centre of the chamber using large aperture parabolic mirrors with focal lengths of 400 mm (for SP1) and 1500 mm (for SP2). At the center

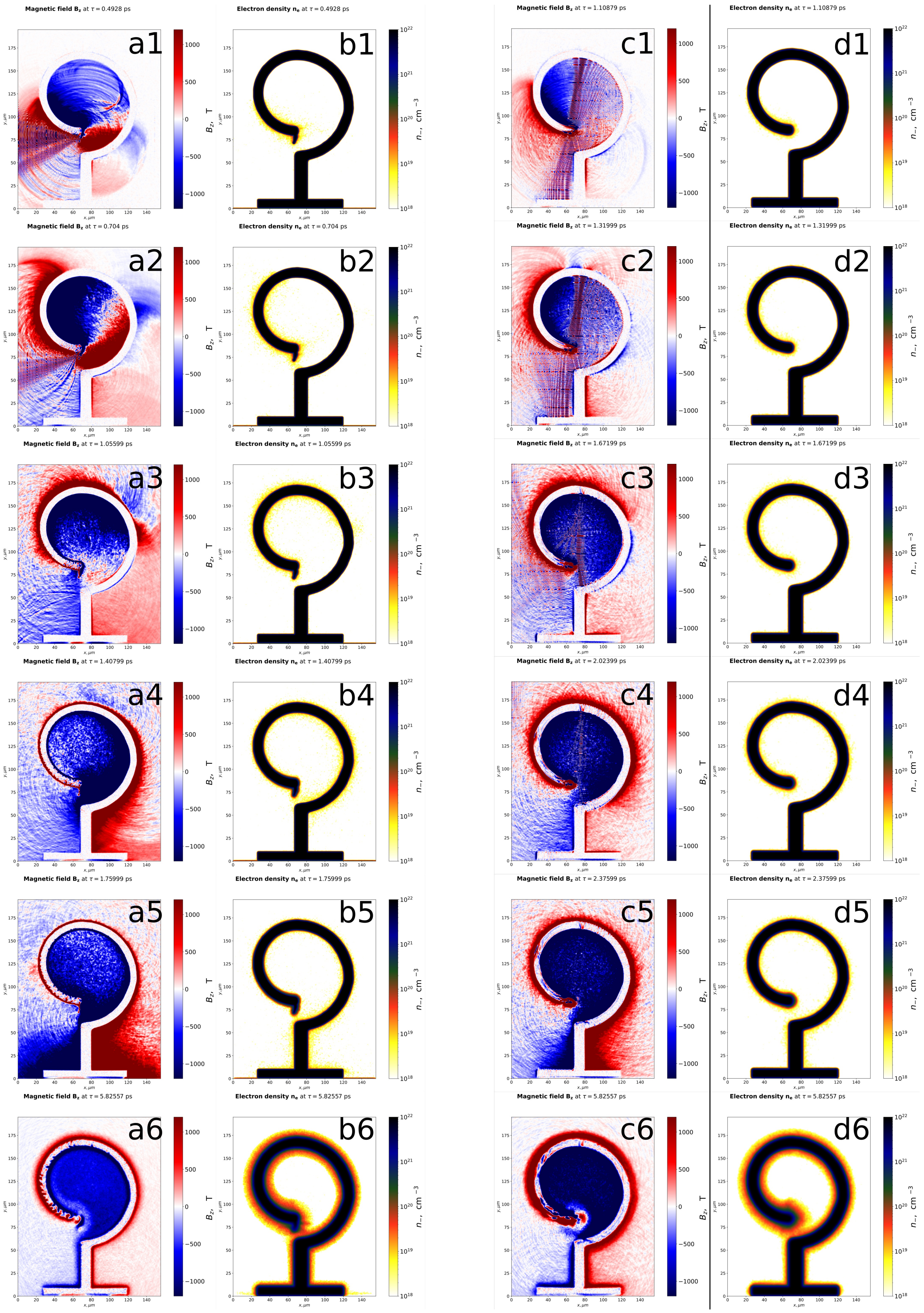

Figure S1: Simulation results with the target size and geometry, close to the real one in the experiment, obtained with the code PICLS. Columns a and c present magnetic field distribution, columns b and d present electron density at different time moments. Results in columns a and b correspond to the interaction with a laser pulse, entering the simulation box from the left, results in columns c and d correspond to the interaction with a model laser pulse, propagating "into" the simulation plane.

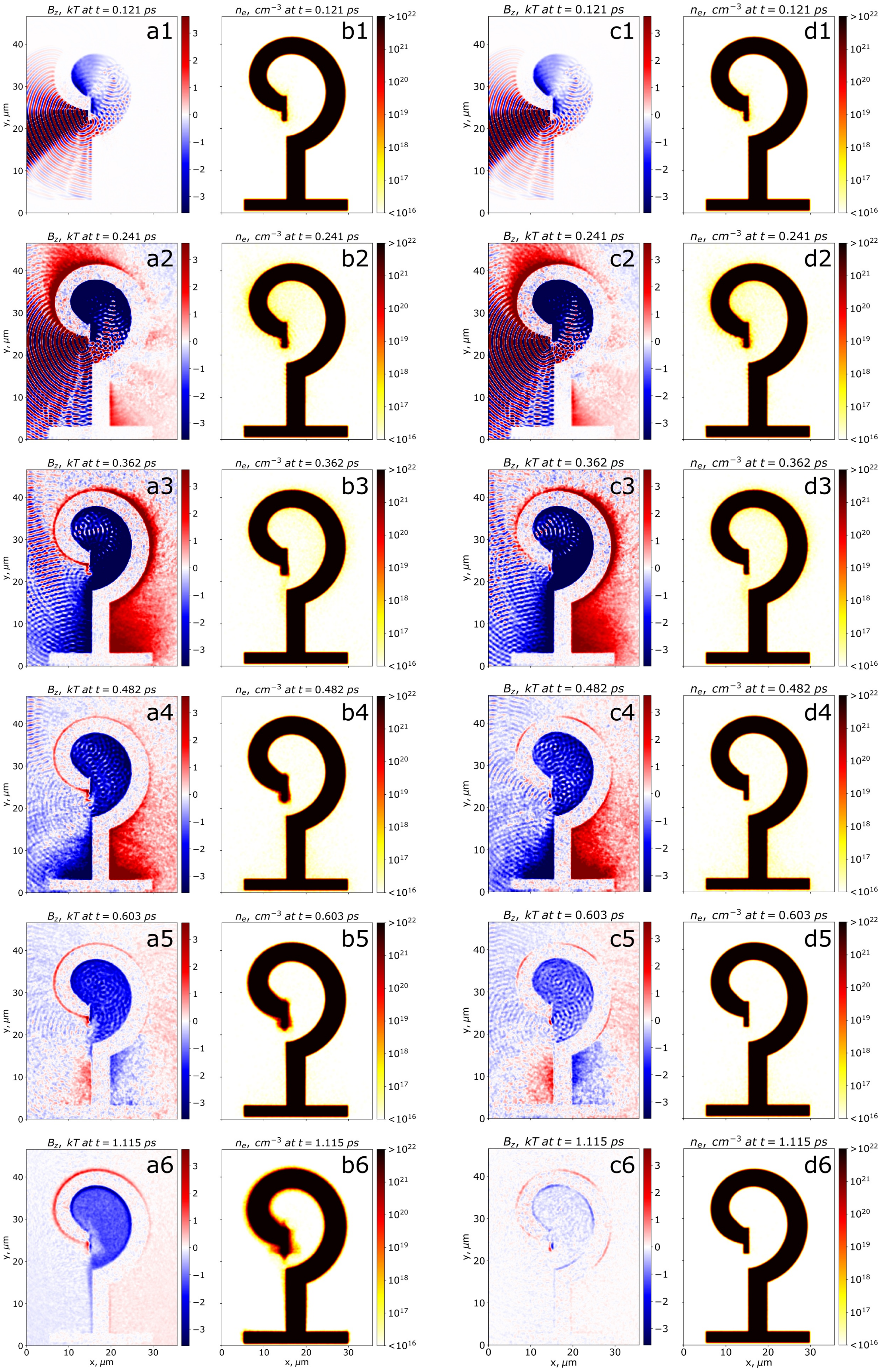

Figure S2: Simulation results with the reduced target size, obtained with the code Smilei. Columns a and c present magnetic field distribution, columns b and d present electron density at different time moments. Results in columns a and b correspond to the 'circuit closure' scenario, results in columns c and d correspond to the 'no circuit closure' scenario.

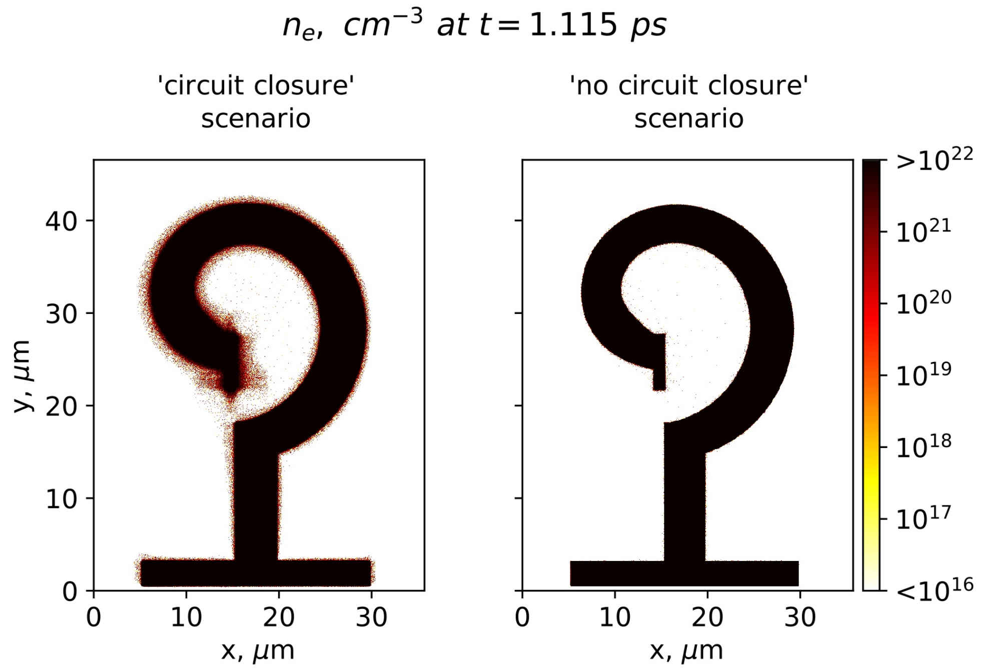

Figure S3: Electron density, obtained with the code Smilei, at $t = 1.115$ ps for 'circuit closure' (left) and 'no circuit closure' (right) scenarios with no spatial averaging applied.

of the vacuum chamber a motorized platform with a studied target, a gold foil and a metallic mesh was installed. The gold foil was used to create diagnostic protons, while the mesh was employed to produce a periodic imprint in the proton beam helpful for analysing the deflection at large distance from the target as well as discerning diagnostic protons from protons emitted from the studied target. A small leakage of SP1 beam was used to create a probe beam line for optical diagnostics - polarimetry and interferometry. An overview of the experimental setup inside the vacuum chamber is presented in Fig. S4.

For precise positioning of the targets at the foci of the laser beams focal spot diagnostic systems were designed and constructed. Each system imaged an object placed at the supposed focal plane onto diagnostic cameras fixed on optical tables outside of the vacuum chamber. During the initial alignment a tip of the needle with a thickness of several microns was used as a test object. Using this test object, sharp images with well-defined edges were obtained, which indicated proper alignment of the focal spot diagnostics. To synchronise the beams in the time domain, a delay line was used for one of the beams. In order to calibrate it, a metallic post was placed at the centre of the vacuum chamber. It was irradiated by periodic pulses of both SP1 and SP2 with 10 Hz repetition rate and at the maximum energy possible during the alignment mode. Each pulse created plasma, the self-emission of which was measured by a fast photodetector connected to an oscilloscope and placed approximately at the bisector of an angle between the optical axes of SP1 and SP2. If one of the beams was delayed relative to the other, a second peak emerged in the signal observed on the oscilloscope. By measuring the time-delay between the two peaks it was verified that the calibration error of the delay line did not exceed 40 ps. More precise calibration was impossible due to insufficient bandwidth of the photodetector as well as the sampling rate of the oscilloscope.

The targets were mounted on glass needles, which, in turn, were placed into plastic holders mounted on motorized platforms with adjustable position and tilt (see Fig. S5). Preliminary alignment of the targets in space and relative to one another was done outside of the vacuum chamber using a 3-axis microscope. The platform with the aligned targets was then connected to the magnetic base at the centre of the vacuum chamber. During this stage, the beams were defocused by shifting the parabolic mirrors along the beam axis to avoid damaging diagnostic cameras or targets themselves. Then the positions of the focal spots on the targets were adjusted. Afterwards, the platform with the targets was moved downwards by a fixed distance, and the beams were brought back to focus. Then the beams were closed with shutters, and the platform with the targets was risen to its initial position. After the vacuum chamber was pumped out, the alignment of the beams was corrected to compensate for the changes of the beam paths in vacuum. Directly before the shot the alignment beam was

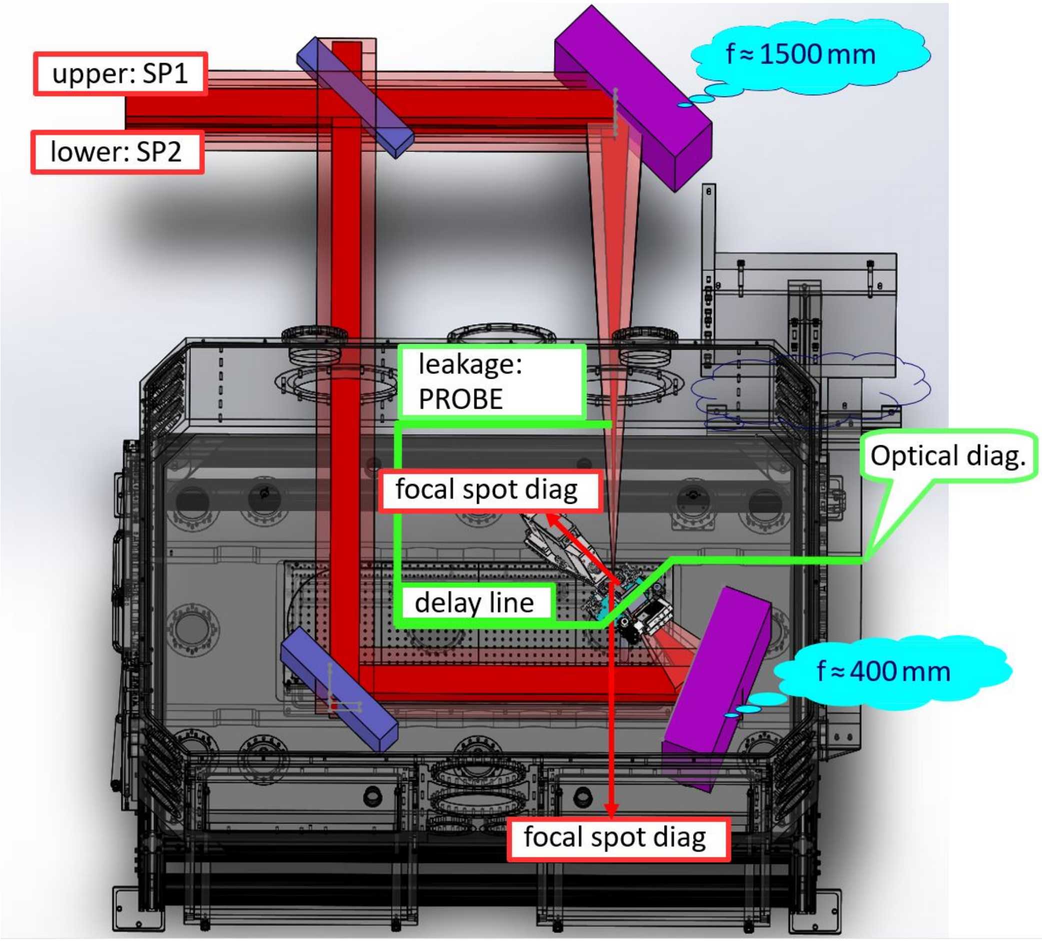

Figure S4: An overview of the experimental setup inside the PHELIX vacuum chamber.

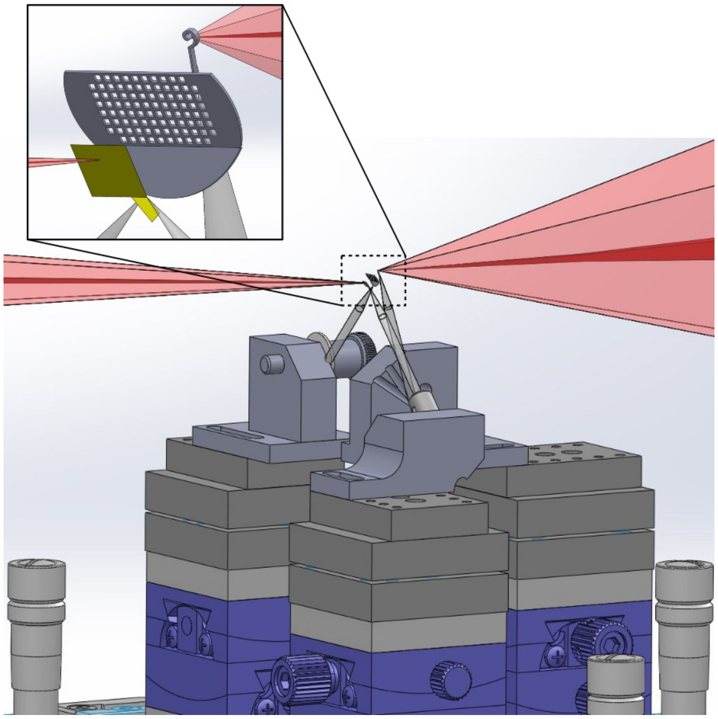

Figure S5: Sketch of the target mounting system.

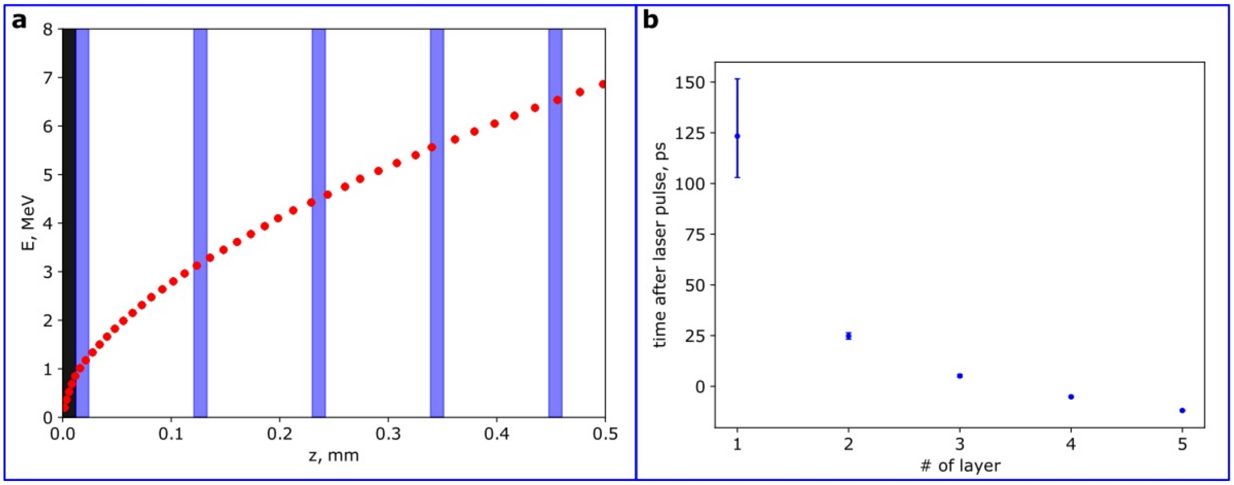

Figure S6: **a:** Stopping ranges for protons of different energies in the first 5 layers of the RCF stack. The 12 μm aluminium foil is marked with black; active layers of Gafchromic HD-V2 films are marked with blue. **b:** Time after the end of the laser pulse, at which protons detected by different layers of the RCF stack probe the fields induced around the spiral target. For layers 3-5 the error bars are smaller than the marker size.

turned off and the shutters were removed, after which standard shot procedure was initiated.

The radiochromic film stack used for measuring the deflection of probe protons in the elctromagnetic fields induced around the spiral target, consisted of a 12 μm aluminium foil filter, cutting the low energy part of the signal ($<$ 1 MeV) containing contributions from heavy ions, and seven Gafchromic HD-V2 films. In order to obtain the energies of protons, producing the signal at different layers of this stack, numerical simulations using the Monte-Carlo code for transport of ions in matter SRIM/TRIM [4] were performed. Thus, stopping ranges for protons of different energies in the RCF stack were calculated. They are shown for the layers, where the proton signal was visible, that is the first 5 layers, in Fig. S6. The active layers, where the proton signal is detected, are marked with blue, the 12 μm alumium filter is marked with black. Substituting the kinetic energies of protons T in the equation $V = c\sqrt{1 - \frac{E_0^2}{(T+E_0)^2}}$, where $E_0 = m_p c^2$ is the rest energy of a proton, we find the velocities of the probe protons, and then, dividing the distance between the TNSA foil and the target $f = 2.95$ mm by the corresponding velocity and taking into account the delay of $t_0 = -96$ ps between the main laser pulse driving the spiral target and the auxiliary laser pulse irradiating the TNSA foil, we obtain the time moment $t = \frac{f}{V} + t_0$ at which protons with the given velocity pass the studied region. For the first 5 layers, where some signal is seen, probing times cover the range from ≈ -10 ps to ≈ 120 ps. The deflection-formed structure is observed on the first 4 layers, however, its quality is sufficient for analysis only for the image on the second layer, formed by the protons probing the field at $24.8^{+1.6}_{-1.5}$ ps after the end of the laser pulse. For faster protons, probing the field at earlier moments of time, the signal is too low, while for slower protons, corresponding to the first layer of the stack, the signal is, on the contrary, too high, which oversaturates the image and obscures most informative patterns in it.

The most informative result in the experiment was produced by proton radiography and a few radiographs were obtained with clearly visible redistribution of diagnostic protons due to deflection in the electromagnetic fields. The presence of strong quasi-stationary magnetic fields, induced around the studied target, can be confirmed qualitatively by comparing the experimental image (see Fig. S7a) with synthetic images that can be obtained via numerical ballistic simulations. If both magnetic and electric field in the interaction region are zero, proton image simply presents a shadow of the target and its stalk, see Fig. S7b. In this case no characteristic 'bubble' void region is observed. If we impose only the electric field, defined by the geometry of the target, charged to a certain potential, the image is also comprised of the shadow of the target, by it is significantly 'bloated' due to positively charged protons deflecting from the surface of the positively charged target, see Fig. S7c. Patterns, similar to the experimental one can only be obtained in the presence of both the electric and the magnetic field, created by the return currents, see Fig. S7d. In this case the characteristic 'bubble' void is formed in the proton beam. Its shape is similar to that of the experimental image and to a certain degree it resembles a cardioid with a slightly pronounced caustic, which for the target orientation in Shot 18 is located in the left part of the main 'bubble'.

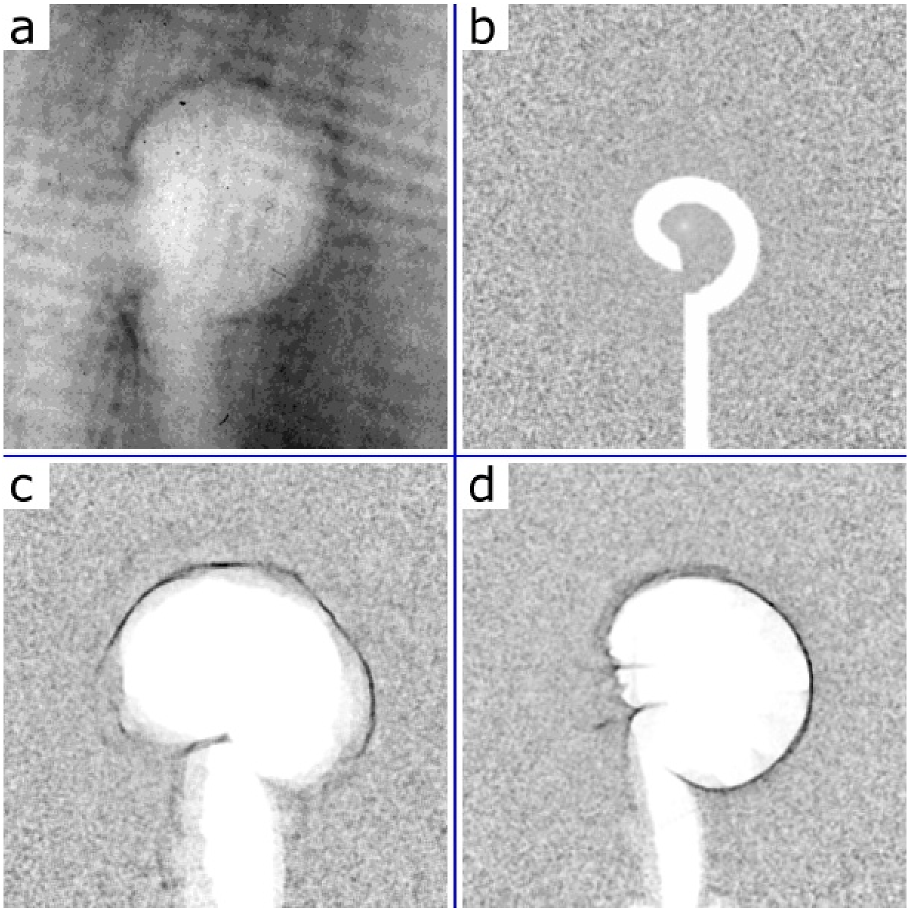

Figure S7: **a:** Experimental proton image, obtained in Shot 18 in the second layer of RCF stack, corresponding to Bragg peak position for ≈3 MeV protons, passing the studied region ≈25 ps after the laser pulse; darker color corresponds to higher proton concentrations. **b:** Synthetic proton image, obtained in ballistic simulations, corresponding to zero electric and magnetic fields in the interaction region. **c:** Synthetic proton image, obtained in ballistic simulations, corresponding to the target electric potential of 100 kV and zero magnetic fields in the interaction region. **d:** Synthetic proton image, obtained in ballistic simulations, corresponding to the target electric potential of 50 kV and the magnetic field of ≈250 T at the target centre.

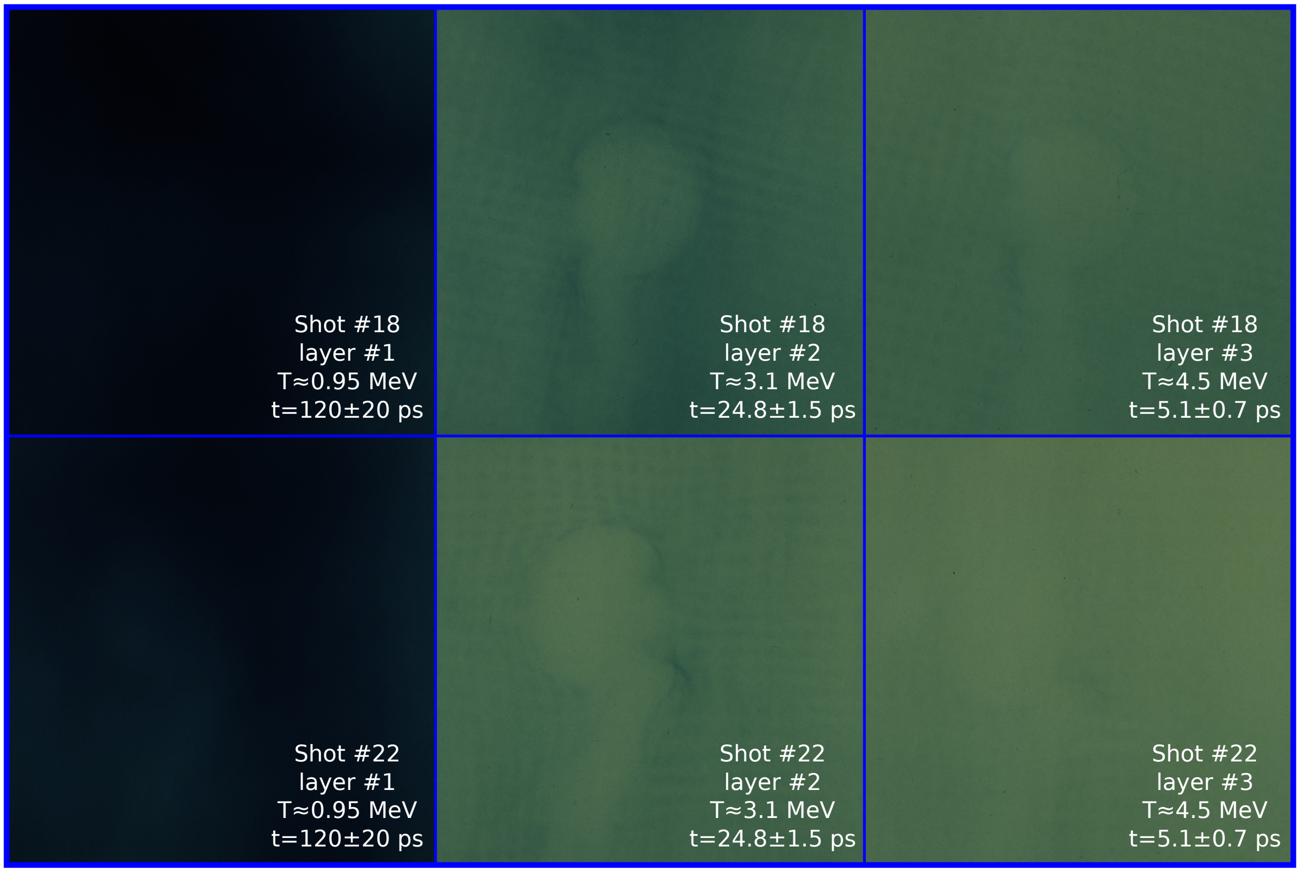

Figure S8: Images, obtained in the first three layers of the RCF stack. In the right lower corner a brief information about each film is displayed - the number of the layer, energy of protons that provide the highest contribution to the signal in it and the moment of time at which these protons pass the target, measured from the end of the laser pulse.

The image presented on Fig. S7a corresponds to one the latest time moment available, that is ≈ 25 ps, which is much greater the the duration of the laser pulse (≈ 0.5 ps). However, a number of other radiographs with non-zero proton signal were obtained in different layers of the RCF stack in the same shot and the other one. They are presented on Fig. S8. Note, that unlike in Fig. S7a, no preprocessing was applied to these images. Their color, brightness and contrast match those of the raw images obtained after scanning the RCFs. As can be seen these radiographs appear to be less informative and useful for the analysis. On RCFs with the data for the earlier time moments the signal is rather dim. In contrast, on the layers with the data for the later time moments the image is for the most part oversaturated due to the very high number of low-energy protons. These factors significantly complicate analysis of experimental images or even make it impossible.

One of the established approaches to retrieving the field parameters from the obtained data is adjusting them in ballistic simulations to provide the best fit to experimental data for a chosen set of geometrical properties of the fast proton pattern. For example, one can adjust the current in the coil to fit the width of the characteristic 'bulb' void [5, 6]. For images similar to Fig. S7 besides the width of the 'bulb' σ it is appropriate to introduce another parameter η, defining the width of the stalk shadow. The latter is important for discerning the effects resulting from the action of the magnetic field and the electric field. As the magnetic field near the stalk is lower than in the cavity, its shadow is mostly defined by the electric potential which enables fitting the latter to experimental values using the parameter η. Obtained results are presented on Fig. S9a. All combinations of the two parameters of the electromagnetic fields that yield similar values of a particular geometric parameter, σ or η lie on a curve that transforms into a band when measurement errors ($\pm 5 - 10$ pixels for 300×300 images) for σ and η are taken into account. Intersection of the two bands corresponds to the estimated ranges of the magnetic field and the electric potential of the target. As can be seen, the intersection region is quite wide, leading to high field retrieval errors. Although, any combination of the parameters from this region yields σ and η similar to experimental values, fast proton patterns themselves may differ. On Fig. S9b and Fig. S9c we provide two

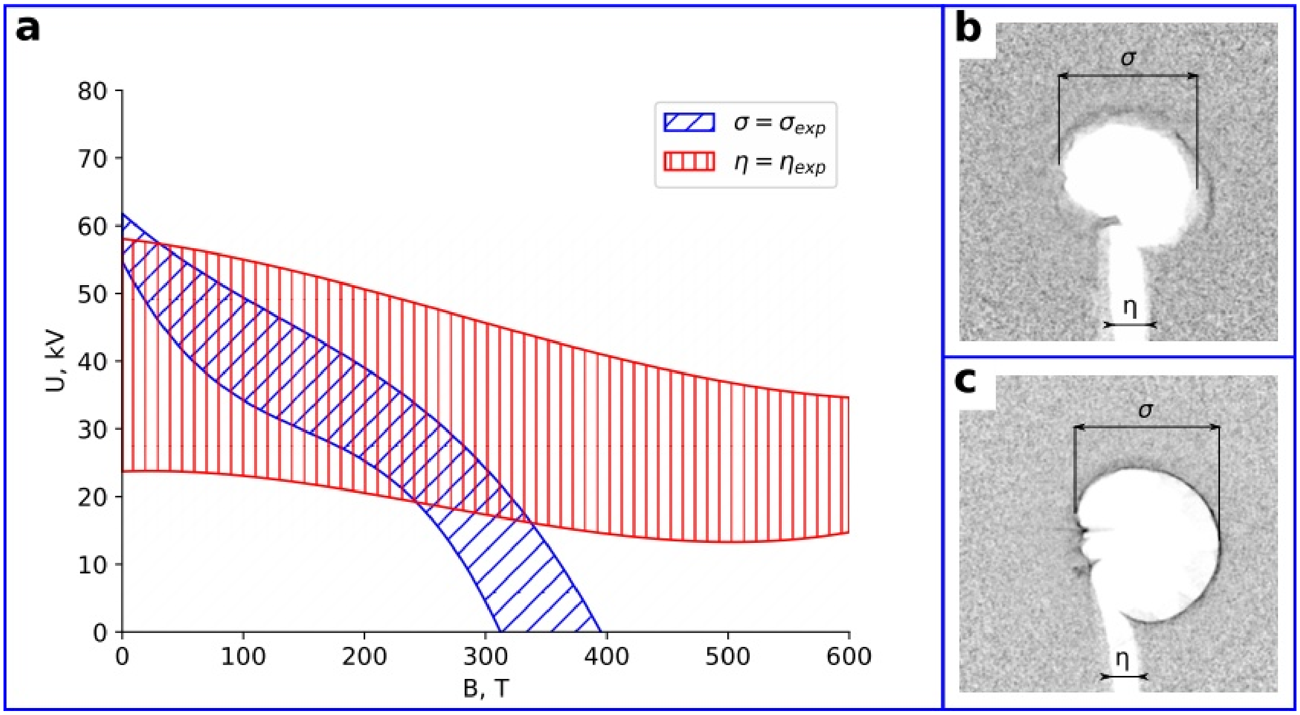

Figure S9: (**a**) Results of parametric fit to the experimental values for parameters σ and η, defined as the width of the upper 'bulb' void region and the width of the stalk shadow, respectively. (**b**) Synthetic radiograph, obtained for $B = 25$ T and $U = 55$ kV, which corresponds to the upper left part of the 'best fit' region. (**c**) Synthetic radiograph, obtained for $B = 265$ T and $U = 30$ kV , which corresponds to the lower right part of the 'best fit region.

simulated images. Parameters of the upper one correspond to the upper left corner of the intersection region, the magnetic field for it is only 25 T and the electric potential is 55 kV. Parameters for the lower one, on the other hand, correspond to the opposite part of the intersection region, with the magnetic field of 265 T and the electric potential of 30 kV. Though, σ and η for both images coincide withing the margin of error, comparison with the experimental data shows that the bottom image appears to reproduce experimental data more accurately. This should be accounted for in the analysis, but it cannot be done with just two geometric parameters, so one has to either introduce even more parameters or use another method that takes into account the whole structure naturally. In this case, cross-correlation and ANN may be more suitable candidates allowing for a more complete and accurate analysis.

The analysis presented in this work is based on processing of the contours of the 'void' regions instead of the images themselves, as their quality is rather different in the experiment and ballistic simulations. The effects which are often irregular and random in experiments cannot be reproduced in numerical simulations: presence of other sources of ionization, i.e. high energy non-diagnostic protons from the studied target, fast electrons, X-ray and gamma, as well as insufficient quality of the diagnostic proton beam and transient behaviour of electromagnetic fields make comparison of the original images unfeasible. In order to reduce the corresponding errors, contours of the 'void' region are considered. The contour retrieval procedure consisted of several steps. First, contrast of both synthetic and experimental images was adjusted to make the extraction procedure more reliable for all synthetic and the experimental images. This was done by modifying the upper and lower limits of the display range, so that a narrower range of values is mapped to 2^8 and 2^{16} possible shades of gray for 8-bit synthetic and 16-bit experimental images, respectively. For all synthetic images, the contrast ratio, i.e. the ratio of the luminosity of the brightest spot to the luminosity of the darkest spot, was improved by a factor of 4, while for substantially dimmer experimental image (see Fig. S8) the contrast ratio was modified by a factor of ~ 10. On the next step, to reduce the noise effects, the images were smoothed by substituting the value of each pixel with the one averaged over the neighborhood of size k. Afterwards, the images were binarized on the basis of an adaptive thresholding algorithm. As the images may still contain significant large-scale noise and have nonuniform illumination, variable thresholds based on local image properties [7] were used to boost the performance of the algorithm. With this approach, the threshold value was calculated for each individual pixel

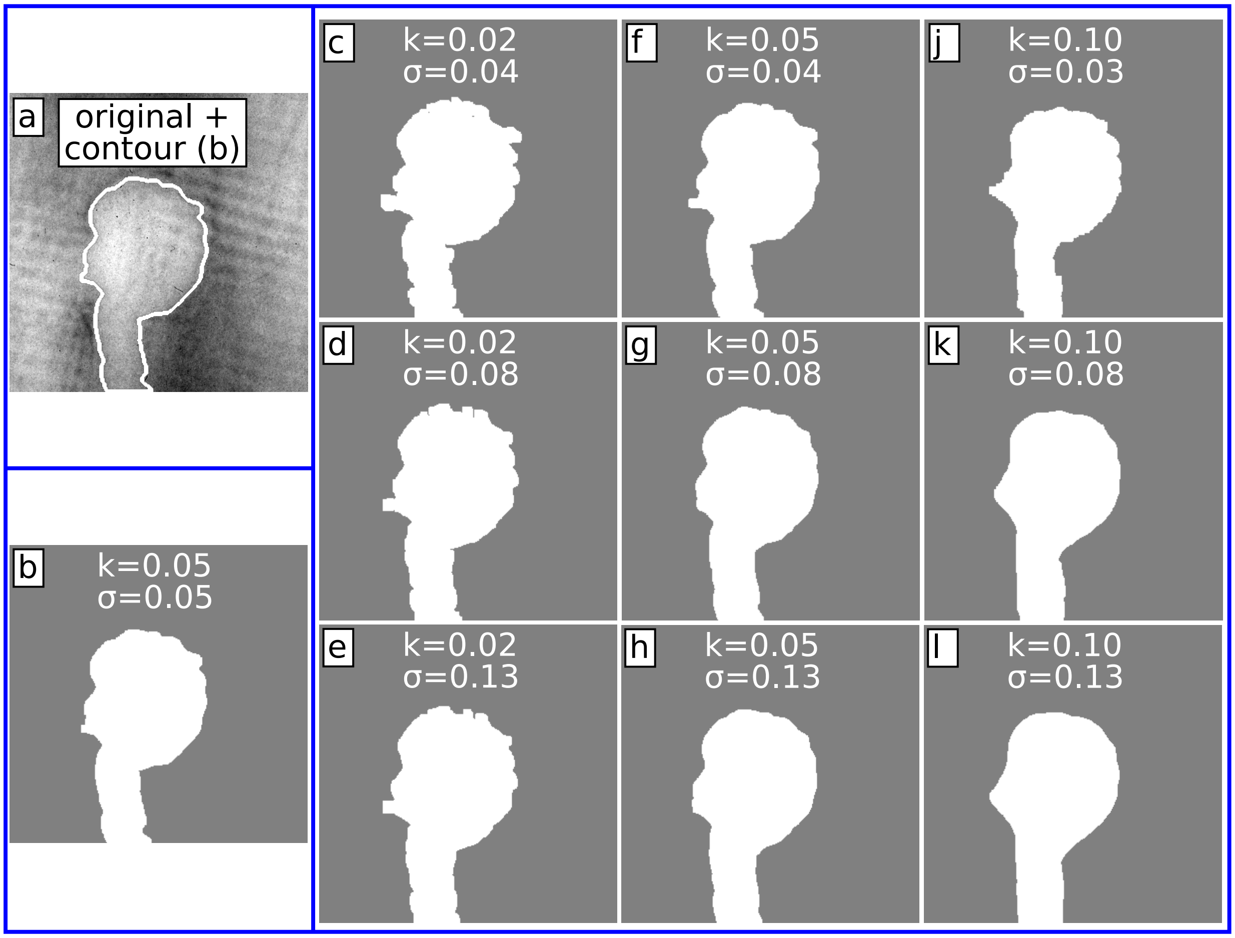

Figure S10: **(a)** Experimental image. **(b)** 'Void' contour corresponding to the intermediate values of k and σ; the skeleton of this contour is imposed on the experimental image **(a)** for easier comparison. **(c)-(l)** 'Void' contours obtained for other different possible combinations of k and σ.

by calculating a gaussian-weighted sum of the neighboring values. In order to find this sum, a convolution with a Gaussian window with standard deviation σ was performed. The procedure was carried out using OpenCV computer vision library functions. An example shown on the library's website [8] demonstrates that adaptive Gaussian thresholding is capable to retrieve image contours in the presence of a strongly non-uniform background correctly. Although this step may not be needed for the synthetic data, it is important for the experimental data, where the illumination level varies for different regions of the image.

The parameters of the contour retrieval algorithm are the size of the averaging kernel k and the characteristic size of the Gaussian threshold filter σ. These parameters were defined as a compromise between preservation of information related to the shape of the 'void' region and appearance of non-informative artefact areas over the main structure. For the images presented in the article, these two key parameters (in fractions of the full image width) were found to meet these conditions around $k = 0.02$ and $\sigma = 0.05$, with variations considered in the range $k = 0.02...0.1$ and $\sigma = 0.03...0.13$ where the final results of the contour extraction look reasonable. Note, that the described algorithm, based on the choice of the parameters (k, σ) in some range, naturally defines the error attributed to the image processing. Indeed, changing parameters of the contour retrieval algorithm may affect the resultant field values due to the changes in the contour shape processed by ANN. Note, that the training set is normally of a better quality and introduce only a minor error, so the main changes come from the experimental data. To estimate the corresponding errors, a set of contours, extracted in the range $k = 0.02...0.1$ and $\sigma = 0.03...0.13$ was considered, see Fig. S10, c-l, the results of processing of these contours with the trained ANN, are summarized in Table S1.

According to the obtained results, presented in Table S1, the mean values of $B_{pred.}$ and $U_{pred.}$ are 222 T and 33 kV, which is within 7 % and 10 % difference, respectively, from the values estimated with initially chosen contour, obtained for intermediate parameters and shown on Fig. S10, b. The overall spread of the values in

$k_{exp.}$	$\sigma_{exp.}$	$B_{pred.}$, T	$U_{pred.}$, kV
0.02	0.04	217.2	42.2
0.02	0.08	207.6	40.6
0.02	0.13	225.3	36.1
0.05	0.04	225.3	32.4
0.05	0.08	246.1	26.7
0.05	0.13	234.2	29.6
0.10	0.03	193.3	34.5
0.10	0.08	225.3	27.2
0.10	0.13	226.7	29.4
Average		222.3	33.2
Δ_{RMS}		14.3	5.3

Table 1: Summary of the results obtained by passing different experimental 'void' contours, corresponding to various combinations of k and σ in the 'optimal' range, through the trained ANN.

root-mean-square error sense is about $\sigma^B_{cont.} = 6$ % for $B_{pred.}$ and $\sigma^U_{cont.} = 16$ % for $U_{pred.}$. The resulting errors for the 95 % confidence level are $\delta^B_{cont.} \approx 14$ % and $\delta^U_{cont.} \approx 36$ %. These errors are of the same order as the training uncertainty calculated from the results of multiple training runs, see Table 1 in the main manuscript, and thus both errors should be taken into account to assess the overall accuracy of the method. Estimating the total error as $\delta_{tot.} = \sqrt{\delta^2_{tr.} + \delta^2_{cont.}}$, where $\delta_{tr.}$ denotes the error related to the ANN training and $\delta_{cont.}$ denotes the error resulting from the contour selection uncertainty, we obtain the values of $\delta^B_{tot.} \approx 23$ % and $\delta^U_{tot.} \approx 46$ % for the relative errors of the magnetic field retrieval and the electric potential retrieval, respectively.

References

[1] Derouillat J. et al. "Smilei: A collaborative, open-source, multi-purpose particle-in-cell code for plasma simulation". In: *Computer Physics Communications* 222 (2018), pp. 351–373.

[2] Y. Sentoku and A.J. J. Kemp. "Numerical methods for particle simulations at extreme densities and temperatures: Weighted particles, relativistic collisions and reduced currents". In: *Journal of Computational Physics* 227.14 (2008), pp. 6846–6861.

[3] Ph Korneev, V Tikhonchuk, and E D'Humières. "Magnetization of laser-produced plasma in a chiral hollow target". In: *New Journal of Physics* 19.3 (2017), p. 033023.

[4] "SRIM – The stopping and range of ions in matter (2010)". In: *Nuclear Instruments and Methods in Physics Research Section B: Beam Interactions with Materials and Atoms* 268.11 (2010). 19th International Conference on Ion Beam Analysis, pp. 1818–1823.

[5] J J Santos et al. "Laser-driven platform for generation and characterization of strong quasi-static magnetic fields". In: *New Journal of Physics* 17.8 (2015), p. 083051.

[6] J. J. Santos et al. "Laser-driven strong magnetostatic fields with applications to charged beam transport and magnetized high energy-density physics". In: *Physics of Plasmas* 25.5 (2018), pp. 1–11.

[7] Rafael C. Gonzalez and Richard E. Woods. *Digital image processing.* Upper Saddle River, N.J.: Prentice Hall, 2008. ISBN: 9780131687288 013168728X 9780135052679 013505267X. URL: `http://www.amazon.com/Digital-Image-Processing-3rd-Edition/dp/013168728X`.

[8] *OpenCV: Image Thresholding.* `https://docs.opencv.org/4.x/d7/d4d/tutorial_py_thresholding.html`. (accessed: 23.05.2022).
